# Pulmonary hypertension: Proteins in the blood

**DOI:** 10.21542/gcsp.2020.7

**Published:** 2020-04-30

**Authors:** Martin Wilkins

**Affiliations:** Imperial College London, Hammersmith Hospital, Du Cane Road, London W12 0NN, United Kingdom

## Abstract

The plasma proteome is rich in information. It comprises proteins that are secreted or lost from cells as they respond to their local environment. Changes in the constitution of the plasma proteome offer a relatively non-invasive report on the health of tissues. This is particularly true of the lung in pulmonary hypertension, given the large surface area of the pulmonary vasculature in direct communication with blood. So far, this is relatively untapped; we have relied on proteins released from the heart, specifically brain natriuretic peptide and troponin, to inform clinical management. New technology allows the measurement of a larger number of proteins that cover a broad range of molecular pathways in a single small aliquot. The emerging data will yield more than just new biomarkers of pulmonary hypertension for clinical use. Integrated with genomics and with the help of new bioinformatic tools, the plasma proteome can provide insight into the causative drivers of pulmonary vascular disease and guide drug development.

## Introduction

The plasma proteome comprises the total complement of circulating proteins in plasma. The Human Peptide Atlas has catalogued over 3,500 proteins that have at least two non-nested uniquely mapping peptides of nine amino acids or more^1^. This is certainly an underestimate, and more will be added as technology develops.

The levels of proteins in plasma varies across an enormous concentration range (∼10^13^ range). Most abundant are proteins with a transport role, while at the other extreme are proteins with signalling properties. Some are secreted while others enter plasma as a result of tissue damage. Collectively these proteins constitute a liquid biopsy that reports on the state of health of tissues. The vast surface area of the pulmonary vasculature makes it likely to be a major contributor to the totality of proteins circulating in blood. Given the risks associated with actually taking biopsies of the lung in pulmonary hypertension, there is particular interest in using the plasma proteome to understand pulmonary vascular disease.

In general, the clinical utility of plasma biomarkers comprises identifying patients at risk of a disease and its outcome, making a diagnosis and following response to treatment. Different biomarkers may be required for each objective. The current view that pulmonary hypertension is an oligogenic phenotype, i.e., the end product of more than one convergent molecular pathway, raises the possibility of more personalised treatments; treatments directed at the particular pathways operative in each patient^2^. Plasma biomarkers may be useful in dissecting the underlying endophenotypes that express this end phenotype to enable the development and deployment of more personalised medicines.

## The current position: Plasma brain natriuretic peptide (BNP), N-terminal proBNP (NT-proBNP) and troponin

Of the available circulating protein markers, only brain natriuretic peptide, measured as BNP-32 or N-terminal proBNP, has made it into clinical practice^3–7^. Plasma BNP and NT-proBNP illustrate the clinical utility of plasma measurements but also the challenges in relying on a single marker to report on a disease. BNP and its cleavage product, NT-proBNP, are secreted from the myocardium and levels correlate with myocardial work^8^. Plasma BNP/NT-proBNP measurements provide a tool for detecting myocardial stress but do not discriminate between possible aetiologies. BNP is elevated in idiopathic PAH^8–13^, PAH associated lung disease^14,15^ and congenital heart disease^16^, chronic thromboembolic pulmonary hypertension^17^, and pulmonary hypertension associated with acute pulmonary embolism^18,19^. BNP levels have to be interpreted in the context of clinical information.

Plasma BNP/NT-proBNP levels correlate with survival; high circulating levels indicate a poor prognosis^9–13^. But these peptides do not report pulmonary vascular disease itself, rather the effect of raised pulmonary artery pressure on the right ventricle. This is not to diminish their importance, as right ventricular function is a major determinant of survival^20^. Nonetheless, there is considerable inter-individual variability; some patients with severe pulmonary vascular disease may have only modestly elevated levels^21^. Other factors such as age, obesity and renal function can affect levels. BNP/NT-proBNP has utility as a non-invasive biomarker for assessing response to treatment in a clinical population. When used for individual patient management, serial measurements are more useful than a single measurement for assessing prognosis; a decrease in NT-proBNP of 15% per year has been reported to be associated with better survival in PAH^21^.

Likewise, plasma troponin measurements reflect myocardial involvement and has little value discriminating pulmonary vascular disease from other causes of myocardial injury^22–24^. Levels correlate with risk of a poor outcome.

## The candidate approach to identifying new protein biomarkers

### Proteins from the myocardium

Other myocardial derived proteins have been investigated for their relationship to right ventricular function and outcome in pulmonary hypertension^25^. Plasma soluble suppression of tumorigenicity (ST2), a member of the interleukin-1 receptor family, is elevated in patients and high levels are related to poor prognosis^26,27^. Endostatin is also secreted from myocardium and levels predict survival^28^. Interestingly, a loss-of-function, missense variant in the gene encoding endostatin, *Col18a1*, was linked to lower circulating protein and independently associated with reduced mortality. Like BNP, ST2 and endostatin are not specific to pulmonary hypertension.

### Vascular-derived proteins

A number of proteins from the vascular wall have been put forward as candidate biomarkers of pulmonary vascular disease^25^. An argument based on the biology of the disease is used to justify their measurement, coupled to measurements in affected tissue.

A widely-held view, supported by the genetics of pulmonary arterial hypertension, is that endothelial dysfunction occurs early in the pathogenesis of pulmonary hypertension. A change in protein synthesis and release from the vascular endothelium might be expected to provide an early sign of pulmonary vascular disease. Progressive structural remodelling of affected vessels which involves all cellular components of the vascular wall provides a broader base for perturbations in protein release, as well as proteins related to thrombosis and inflammation.

The success of endothelin antagonists as a treatment for PAH provides validation for an integral role for endothelin in the underlying pathology^29^. Plasma endothelin-1 levels are elevated in PAH and correlate with haemodynamic measurements^30–33^, but levels are influenced by endothelin receptor antagonists that block the ETB receptor^34^.

Angiopoietin-2 is synthesised by vascular endothelial cells and acts as an antagonist of angiopoietin-1 signalling^35^. Increased expression has been reported in plexiform lesions in PAH, but not in unaffected adjacent arterioles within the same IPAH lung tissue samples. Elevated circulating levels correlate with haemodynamic measurements and elevated circulating levels predict poor survival in idiopathic PAH.

Other proteins synthesised by vascular endothelial cells, such as von Willebrand factor, GDF-15 and thrombospondin-1, are also elevated in PAH and correlate with poor survival^36–41^. The soluble form of the vascular endothelial growth factor receptor-1 (sFlt-1) and placental growth factor (PIGF) are also elevated in PAH; there is no consensus whether sFlt-1 associates with risk^42^. Soluble ICAM-1 is elevated in children with PAH^43^ and raised levels of other adhesion molecules have been reported in other presentations of pulmonary hypertension, such as systemic sclerosis and sickle cell disease^44^.

Beyond endothelial cells, osteopontin, produced by a number of cell types, is increased and levels relate to clinical severity and outcome^45^. Consistent with the pronounced matrix remodelling associated with many presentations of pulmonary hypertension, patients with high circulating levels of N-terminal propeptide of procollagen III (PIIINP), matrix metalloproteinase-9 (MMP-9) and tissue inhibitor of metalloproteinase I (TIMP-1) have a significantly worse survival than patients with lower (≤median) levels^42,46,47^.

### Inflammatory proteins

PAH is an inflammatory condition and associated with elevated circulating cytokines and chemokines. Among these are tumor necrosis factor (TNF), many interleukins (IL) including IL-1b, IL-4, IL-6, IL-8, IL-10, IL-12p70, and IL-13, fractalkine (CX3CL1), RANTES (CCL5), monocyte chemotactic protein-1 (MCP-1; CCL2), and interferon g-induced protein 10 (IP-10; CXCL10).^48–52^ Kaplan-Meier analysis showed that levels of IL-6, 8, 10, and 12p70 predicted survival in patients^49^.

Unsupervised machine learning has also been applied to a proteomic dataset from a heterogenous cohort (n = 281) of patients with PAH, comprising equal numbers of patients with idiopathic PAH and PAH associated with connective tissue disease, and a smaller number of patients where PAH was related to toxins or congenital heart disease^53^. A panel of 46 cytokines, chemokines, and growth factors were measured using a multiplex immunoassay. Four PAH clusters with distinct proteomic immune profiles were identified.

Interestingly, PAH clinical subtypes, comorbidities, and medications were similar across the clusters but the clusters differed by risk. Cluster 1, containing tumor necrosis factor-related apoptosis-inducing ligand (TRAIL), C-C motif chemokine ligand 5 (CCL5), CCL7, CCL4 and macrophage migration inhibitory factor (MIF), was associated with the highest risk. Cluster 3, containing interleukins-12 (IL-12), IL-17, IL-10, IL-7 and vascular endothelial growth factor (VEGF), was linked with the lowest risk. Cluster 2, with low cytokine levels similar to controls, and cluster 4, comprising IL-4, IL-6, IL-8, CCL11 and platelet-derived growth factor beta (PDGF- β) demonstrated intermediate risk. These findings were replicated in a smaller validation cohort (n = 104). The use of blood cytokine profiles to distinguish PAH immune phenotypes with differing clinical risk that are independent of World Health Organization group 1 subtypes could provide a framework to examine patient responses to emerging therapies targeting immunity.

## The agnostic approach based on plasma proteome assays

The alternative to selecting candidates for investigation is the shotgun approach and new technologies has made this accessible^54^. These use high-throughput multiplexed assays that can provide measurements, relative or absolute (depending upon the assay), of many proteins in one small plasma aliquot without prefractionation. Each has its limitations^55^ but early results demonstrate the value of agnostic screening that go beyond simply biomarker discovery and validation^56–61^.

**Figure 1.**
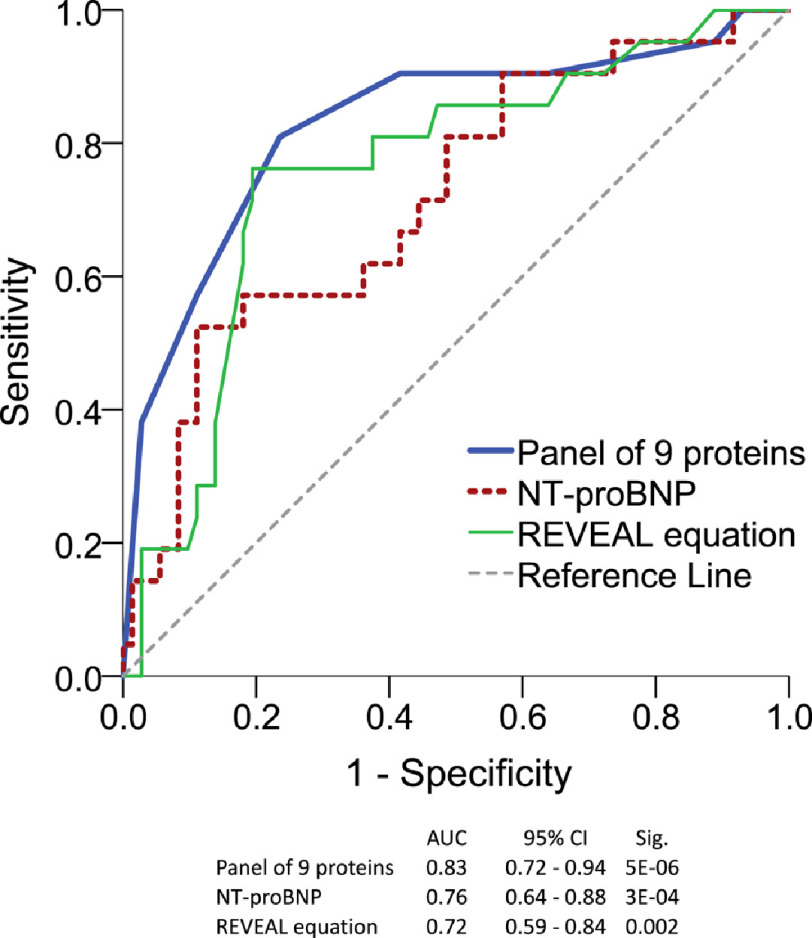
Comparison of 9-protein panel with NT-proBNP and REVEAL score. Prediction of survival in cohort of patients with idiopathic or heritable PAH. (Based on data from Reference^64^).

The SomaLogic platform has now been used in a number of cardiovascular studies^54,61–63^. It is an aptamer-based platform and an early version offered results on 1,129 proteins in a single aliquot. This was used in a cross-sectional study to analyse samples from 397 patients with idiopathic or heritable PAH from 3 expert European centres^64^. Forty proteins detected by the assay were linked to survival in a discovery and validation analysis. Of these, 20 were prioritised by random sampling analysis and 14 proved prognostic independent of NT-proBNP. Independent assays (ELISA and Luminex) were available for 9 of the 20 proteins and showed good correlation with the aptamer assay results. A protein panel score was then devised, based on thresholds derived from Receiver Operating Characteristic (ROC) curves, such that each of the 9 proteins could be scored for each patient and the composite score used to quantify individual risk of death. The 9-protein panel improved risk estimates, providing complementary information to a well-accepted clinical risk equation (Figure 1).

The 9 proteins in the panel comprised ST2, tissue inhibitors of metalloproteinases (TIMP-1 and TIMP-2), plasminogen, apolipoprotein-E (ApoE), erythropoietin (EPO), complement factor H and factor D, and insulin-like growth factor binding protein-1 (IGFBP-1). These proteins cover myocardial stress, collagen turnover, fibrinolysis, inflammation, iron status and factors that influence cell proliferation. This multiplex biomarker approach may be expected to better capture and report on the activity of the vascular pathology than BNP alone, but may also better stratify PAH patients according to the underlying endophenotype(s).

There have been few attempts to date to compare the plasma proteome across different presentations of pulmonary hypertension. One study^65^ used an immunoassay platform to measure 28 proteins associated with receptor tyrosine kinase signalling in 152 patients with different presentations of pulmonary hypertension; PAH, chronic thromboembolic disease, pulmonary hypertension due to diastolic heart failure (preserved ejection fraction), pulmonary hypertension due to systolic heart failure (reduced ejection fraction) and heart failure without pulmonary hypertension, as well as healthy control subjects. Plasma proto-oncogene tyrosine-protein kinase receptor Ret (RET) levels were decreased in PAH compared with all disease groups and controls. Plasma tyrosine-protein kinase MER, vascular endothelial growth factor (VEGF)-A, VEGF-D, placental growth factor, amphiregulin, hepatocyte growth factor and transforming growth factor- α were all increased and decreased VEGF receptor-2 and epidermal growth factor receptor levels were decreased compared with controls.

Multi-centre collaboration is often needed to acquire the number of samples needed for a study in PAH, particularly when the focus is on incident cases to minimise the impact of treatment. Indeed, recruiting patients from different centres is preferable to understand how results apply in the broader population. An issue with multi-centre studies is variation is sample collection. One of the first observations when data is subject to principle component analysis is the origin of the samples by centre. Use of a standardised operating protocol by all centres is essential to minimise heterogeneity by centre leading to false positive and negative associations.

## Network analysis

Mapping the proteins that differ in levels onto a protein-protein network can offer deeper insights into the pathways that contribute to pathology. For example, a pathway can highlight proteins that may not be part of the assay platform but are clearly involved as they link proteins that were measured and changed.

A panel of proteins that emerged as prognostic from the SomaLogic platform^64^ has been mapped to the consolidated interactome, a network of functional associations between proteins curated from the literature and other sources^66,67^. From this, a network of 13 proteins disturbed in PAH patients were interconnected through 18 protein-protein interactions^68^. This network was heavily populated by complement proteins from the complement alternative pathway. Further investigation of this PAH complement network in a PAH cohort identified two patient clusters which differed in rate of all cause mortality. This link with survival suggests that the alternative complement pathway influences the course of PAH and could be a therapeutic target.

## Using protein quantitative trait loci (pQTLs)

Plasma protein levels, like tissue expression, is influenced by genetic factors. Integrating the plasma proteome with genetic data has identified a large number of genetic associations with circulating proteins^56–60^. These protein quantitative loci –pQTLs –are useful instruments in Mendelian randomisation analysis^54^, whereby genetic factors can be linked to disease via specific proteins. Mendelian randomisation is a method that sits between observational studies (very weak evidence of causality) and interventional studies (strong evidence of causality). In this instance, it assumes that the effect of a genotype on the phenotype is mediated solely via the protein. It has the potential to link biomarkers to PAH by causation and so prioritise further biological study.

The full potential of this approach in PAH has yet to be explored but some interesting opportunities are emerging. In addition to insights into the cause, pQTLs can be used to inform drug development. A pQTL for circulating platelet-derived growth factor receptor beta (PDGFRb) is highly significant. The “minor allele” frequency (at least in Europeans) for this pQTL is around 45%. PDGFR is one of the targets for imatinib. This drug has been investigated in a Phase 3 study (IMPRES) in PAH with some patients demonstrating an improvement in haemodynamic and functional measurements. Indeed, some patients have shown a dramatic improvement when other interventions have failed. Adverse effects with the doses used have stalled further investigation of the drug in PAH. A working hypothesis is that the pQTL for PDGFRb might identify patients who might respond to the drug, perhaps at lower doses than those used in IMPRES, and so improve the benefit/harm ratio. A genetic biomarker-driven study whereby the patient response to imatinib is interpreted according to genotype is about to start.

## Conclusion

The plasma proteome is still under-explored. Progress has been made using samples from a mixture of incident and prevalent patients, taken opportunistically. And the focus has been on PAH. There is more to be gained from a better co-ordinated strategy that engages the broader presentation of pulmonary hypertension using a standard operating protocol, a common assay platform and follow up samples. This will be essential if the aim is to develop a diagnostic. But it is also important if we are to progress from a clinical classification of pulmonary hypertension to one informed by molecular profiles. The plasma proteome can contribute to a deeper understanding of pulmonary vascular disease that will inform new drug development and personalised medicine.
